# Optical and Electrochemical Properties of a Nanostructured ZnO Thin Layer Deposited on a Nanoporous Alumina Structure via Atomic Layer Deposition

**DOI:** 10.3390/ma17061412

**Published:** 2024-03-20

**Authors:** Ana L. Cuevas, Antonia Dominguez, Javier Zamudio-García, Victor Vega, Ana Silvia González, David Marrero-López, Victor M. Prida, Juana Benavente

**Affiliations:** 1 Unidad de Nanotecnología, Centro de Supercomputación y Bioinnovación, Servicios Centrales de Investigación, Universidad de Málaga, 29071 Málaga, Spain; analaura.cuevas@uma.es (A.L.C.); adhuertas@uma.es (A.D.); 2 Department of Energy Conversion and Storage, Technical University of Denmark, 2800 Lyngby, Denmark; javzam@dtu.dk; 3 Laboratorio de Membranas Nanoporosas, Servicios Científico-Técnicos, Universidad de Oviedo, 33006 Oviedo, Spain; vegavictor@uniovi.es; 4 Departamento de Física, Facultad de Ciencias, Universidad de Oviedo, 33007 Oviedo, Spain; gonzalezgana@uniovi.es (A.S.G.); vmpp@uniovi.es (V.M.P.); 5 Departamento de Física Aplicada I, Facultad de Ciencias, Universidad de Málaga, 29071 Málaga, Spain; marrero@uma.es

**Keywords:** nanoporous alumina, ZnO coating, microstructural and optical characterizations, impedance spectroscopy

## Abstract

This study explores the optical and electrochemical properties of a ZnO coating layer deposited on a nanoporous alumina structure (NPAS) for potential multifunctional applications. The NPAS, synthesized through an electrochemical anodization process, displays well-defined nanochannels with a high aspect ratio (~3000). The ZnO coating, achieved via atomic layer deposition, enables the tuning of the pore diameter and porosity of the NPAS, thereby influencing both the optical and electrochemical interfacial properties. A comprehensive characterization using photoluminescence, spectroscopy ellipsometry and impedance spectroscopy (with the sample in contact with NaCl solutions) provides insights into optical and electrochemical parameters, including the refractive index, absorption coefficient, and electrolyte–ZnO/NPAS interface processes. This research demonstrates potential for tailoring the optical and interfacial properties of nanoporous structures by selecting appropriate coating materials, thus opening avenues for their utilization in various technological applications.

## 1. Introduction

The fabrication of materials with reduced (nanometric) dimensions, such as nanoparticles, nanopores, nanorods or nanowires and nanotubes, has emerged as an interdisciplinary research field with applications in different areas. Particularly, nanoporous structures (NPSs) fabricated with ceramic oxides offer numerous advantages, including high hardness, thermal and chemical resistance, as well as relatively low cost. Among these, nanoporous alumina structures (NPASs) synthesized by a two-step electrochemical anodization process [1,2] exhibit highly ordered straight cylindrical nanochannels with uniform pore sizes, providing NPASs with high aspect ratios (a large surface area per unit volume). The pore radii (ranging from 10 nm to 200 nm) and pore density (porosity) are finely controlled and strongly depend on specific anodization conditions such as the electrolyte and pH, anodization voltage and temperature) [3]. The practically ideal highly self-ordered nanoporous geometry of NPASs guarantees their potential application in nanofluidics, drug delivery or as nanofilters [4,5,6]. Additionally, they exhibit interesting optical characteristics determined by pore size and the growing oxide layer thickness, behaving as photonic crystals and displaying photoluminescence [7,8,9,10]. Both the geometrical parameters and surface characteristics of the NPASs can be changed through appropriate surface modifications. Among other surface modification processes, atomic layer deposition (ALD) is a well-known technique allowing for the deposition of coating layers of different materials (ceramic oxides, metals, nitrides, sulfides, etc.) onto different substrates [11,12,13,14]. This technique is based on the sequential exposure of a substrate to different gaseous precursors, which undergo surface-limited reactions at the surface of the substrate, thus leading to uniform, conformal coating with atomic-level thickness control. Therefore, ALD is particularly suitable for surface coating and functionalization of NPASs due to their complex 3D nanostructuring. In fact, the effect of surface coating NPASs with one or two layers of different ceramic oxides (SiO_2_, TiO_2_, Fe_2_O_3_, etc.) using the ALD process on their optical properties performed has already been reported [15,16]. Similarly, the effect of pore-size/porosity and surface layer material on ion diffusion through these nanoporous alumina-based structures (NPA-bSs) as well as on the NPA-bSs/electrolyte solution interface, has already been analyzed [16,17,18]. These changes seem to be associated with variations in the hydrophilic/hydrophobic features of samples surfaces and/or the charge of the NPA-bSs walls. Consequently, a reduction in the diffusion of ions, or even the almost total exclusion of co-ions from the nanopores (cations/anions depending on the positive/negative sign of charges) was obtained [5,19,20,21]. Such behavior is of significant interest for applications involving the transport of aqueous electrolyte solutions through nanofilters or membranes. 

In this work, we investigate the optical and electrochemical properties of an NPAS coated with a ZnO layer using the ALD technique (Al/ZnO sample). Surface coverage of different kinds of substrates by thin films or uniform nanoparticles is currently of great importance in numerous areas (energy storage, catalysis, solar energy conversion, microelectronic devices, etc. [22,23]). ZnO was selected for layer coating due to its favorable chemical and physical properties, including high chemical stability, a broad range of radiation absorption, a high electrochemical coupling coefficient, photoluminescence and anti-biofouling behavior [24,25,26,27,28,29]. These properties are of widespread interest for applications in diverse fields, particularly in high-tech sectors such as solar cells, biomedical, sensing and water treatment using micro-nanoporous membranes [30,31,32,33]. The geometrical parameters and hydrophobic character of the Al/ZnO nanoporous layer were estimated by SEM micrographs and contact angle measurements, respectively. Additionally, its optical characteristics were determined by analyzing the photoluminescence spectra and spectroscopy ellipsometry experimental methods. The latter technique provides information on the wavelength dependence (in the range between 200 nm and 2000 nm) of the refractive index, the absorption coefficient, and the dielectric constant (real and imaginary parts) of the analyzed samples, as well as for the estimation of layers thickness. Spectroscopy ellipsometry has also been recently employed in the characterization of biosensors for the detection of molecules with clinical interest [34,35]. 

On the other hand, electrochemical impedance spectroscopy measurements were performed with the Al/ZnO nanoporous film in contact with NaCl solutions of different concentrations. By obtaining these measurements, we aimed to gather information on electrical properties associated with both the Al/ZnO sample and the electrolyte–Al/ZnO interface, the latter being directly related to membrane fouling [36,37], a significant problem that occurs in the treatment of liquid mixtures by membrane processes [38]. Consequently, the findings of this analysis might be of great interest for the application of the Al/ZnO nanoporous film as a nanofilter or membrane.

## 2. Materials and Methods

### 2.1. Material Preparation

The nanoporous alumina structure (NPAS), obtained by the two-step anodization method as detailed elsewhere [1], was subsequently coated with a thin layer of ZnO using the ALD technique. This nanoporous structured sample will hereafter be referred to as Al/ZnO. The NPAS used as substrate was obtained using high-purity aluminum discs (Al 99.999%, Goodfellow, UK; 25 mm in diameter and thickness of 0.5 mm) and an aqueous solution of sulfuric acid (0.3 M) was used as the electrolyte, with an anodization voltage of 25 V at 0–1 °C for 24 h. An aqueous mixture of CrO_3_ and H_3_PO_4_ (at room temperature for 48 h) was used for the selective chemical etching of the oxide layer grown during the first anodization step, as previously described [16]. The geometrical parameters of the NPAS substrate (aspect ratio higher than 2000) are as follows: pore radii of (14 ± 2) nm, porosity of 16% and a thickness layer of around 60 µm.

The ZnO coating layer on the Al/ZnO sample was deposited using a Savannah 100 thermal ALD reactor from Cambridge Nanotech (Waltham, MA, USA), working in exposure mode to allow gaseous precursors to effectively diffuse throughout inside the alumina pore channels. Diethylzinc (C_4_H_10_Zn) at 20 °C and water (at 60 °C) were used as precursors for the deposition of the ZnO conformal coating layer. The substrate temperature was maintained at 200 °C, and high-purity Ar was employed as the carrier gas, as described in a previous work [19]. A total of 44 ALD cycles were programmed to adjust the thickness of the ZnO layer to around 4–5 nm, according to the expected growth rate for ZnO thin film previously reported in the literature [39]

### 2.2. Scanning Electron Microscopy, X-ray Diffraction and Contact-Angle Measurements

Surface characterization of both the NPA and Al/ZnO samples, as well as the estimation of their respective morphological parameters such as the pore radii and porosity, were conducted using scanning electron microscopy (SEM) in a JEOL JSM-5600 microscope (Mitaka, Japan), equipped with a tungsten electron gun and operated at an acceleration voltage of 20 kV. Due to the electrically isolating nature of the former nanoporous alumina, samples were coated with a thin Au metallic layer using magnetron sputtering deposition (Polaron SC7620) for 180 s at 20 mA prior to SEM observation, thereby avoiding charging surface effects. The acquired images were further analyzed by using ImageJ v1.8.0 software [40].

The crystalline structure of the Al/ZnO sample was examined using X-ray diffraction (XRD) with a PANalytical Empyrean diffractometer in the 2θ range from 10 to 80° with CuK_α_ radiation. The analysis of the ZnO coating layer deposited onto the nanoporous alumina substrate was performed using the grazing incidence technique. Crystalline phase identification and pattern diffraction analysis were carried out using the X’Pert HighScore Plus software v3.0e [41].

The hydrophobic/hydrophilic characteristics of the ALD deposited Al/ZnO nanoporous thin film were determined from contact-angle measurements. The measurements were performed with distilled water using a Teclis T2010 instrument (Bordeaux, France). equipped with a video system for a period of two minutes to determine an averaged value from three different measurements.

### 2.3. Optical Characterization

The photoluminescence spectrum was measured at room temperature using a photoluminiscence microscope from HORIBA Scientific (LabRam PL Microscope, Horiba Scientific, Tokyo, Japan) equipped with a laser excitation light λ_ex_ = 325 nm and beam power of 0.028 mW. 

Spectroscopic ellipsometry (SE), a technique commonly used to characterize single layers or a small number of well-defined layers that are optically homogeneous and isotropic, measures the change in light polarization upon reflection or transmission by determining spectroscopic Ψ and Δ angles. These angles are related to the amount of reflected light polarized in the perpendicular and parallel planes with respect to the incidence light planes [42]. SE measurements were carried out with unsupported samples using an ellipsometer (Sopra-Semilab GES-5E, Budapest, Hungary), which operates in a wavelength range between 200 nm and 1600 nm. Measurements were acquired at two incident angles: Φ_o_ = 65° and Φ_o_ = 75° to difference deeper from more superficial effects. Data analysis and fittings were performed with WinElli software v.2.2 (Sopra-Semilab). 

### 2.4. Electrochemical Characterization of the Electrolyte–Al/ZnO Nanoporous Film System

Electrochemical impedance spectroscopy (EIS) measurements for electrical characterization of the Al/ZnO nanoporous film were conducted in a dead-end electrochemical cell (see Figure S1 in the Supplementary Information), using a frequency response analyzer (FRA, Solartron 1260, Hampshire, UK). The frequency (f) ranged between 1 Hz and 10^7^ Hz with a maximum ac voltage of 10 mV. Measurements were performed with the Al/ZnO sample in contact with two NaCl solutions of the same concentration (Pt-electrode/solution (c)/sample/solution (c)/Pt-electrode) at different solution concentrations: c = 10^−3^, 2 × 10^−3^, 5 × 10^−3^, 10^−2^ and 2 × 10^−2^ M. The electrode size was larger than the sample size to ensure the uniformity of the electric field. Impedance data were analyzed by equivalent circuit models [43] and Distribution of Relaxation Times (DRT) with ZView (Scribner Associates Inc., Southern Pines, NC, USA) and DRT tools software [44], respectively.

## 3. Results

### 3.1. Microstructural Properties

**Figure 1:**
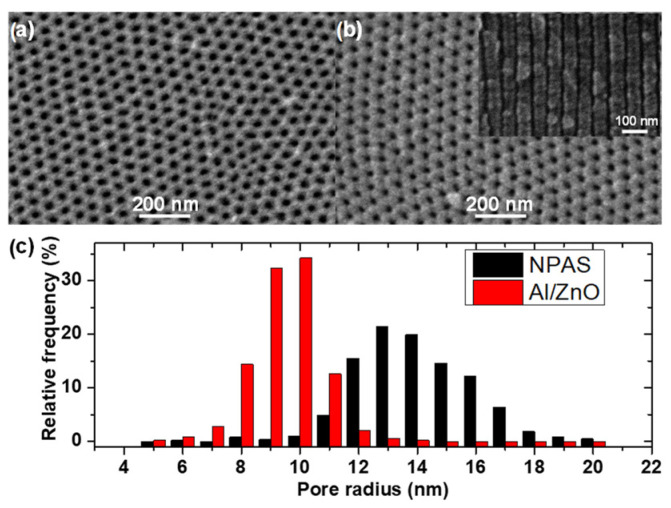
Top-view surface SEM images of (**a**) as produced NPAS and (**b**) Al/ZnO sample after being ALD-coated with ZnO. The inset in (**b**) is a SEM cross-section image of the pore channels. (**c**) Pore radii distribution obtained from SEM images in (**a**,**b**).

Figure 1 displays the top-view surface SEM images of the NPAS template prior (a) and after (b) being coated with a ZnO layer by ALD. The highly ordered pores arrangement, as well as the reduction in pore size, becomes evident from the comparison of both images. The straightness of pore channels is confirmed in the SEM cross-section view shown as inset in Figure 1b. Furthermore, the pore radius distribution was evaluated by image analysis of both samples, as shown in Figure 1c. These results yield average pore radius values r_p_ of 14 ± 2 and 9 ± 2 nm for NPAS and Al/ZnO samples, respectively. These values indicate that the thickness of the ZnO layer deposited by ALD onto pores of the NPAS sample is approximately 5 nm, confirming the deposition rate reported in the literature [39] and estimated in the Experimental Section. The interpore distance, D_int_, remains unchanged after the ZnO deposition and takes a value of 65 nm. Taking into account the hexagonal symmetry, the porosity can be calculated as (Θ = (2π/√3) × (r_p_/D_int_)^2^ [2], achieving a value of approximately 7% for the Al/ZnO sample. Consequently, the ZnO coating layer deposited by ALD onto the NPAS substrate reduces its pore size by around 35% and the porosity by 55%. 

**Figure 2:**
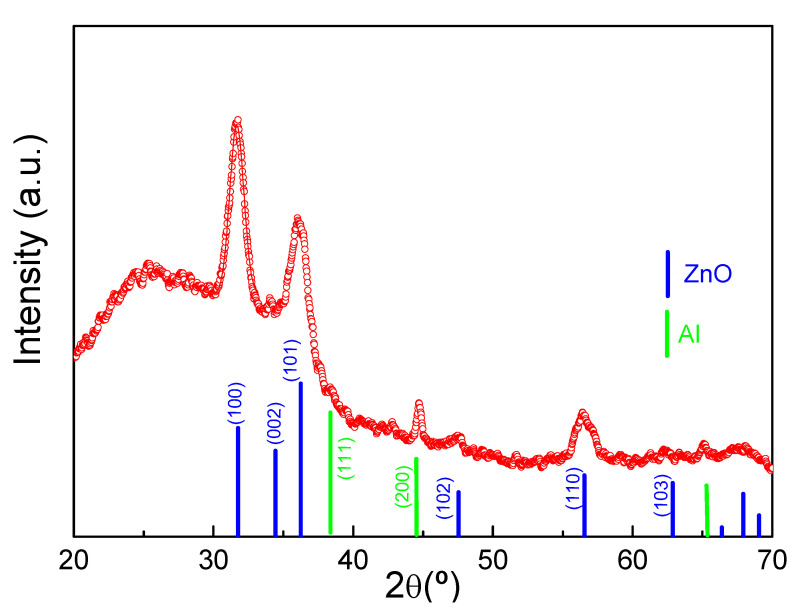
Grazing incidence XRD pattern of Al/ZnO sample. The theoretical XRD pattern of the hexagonal ZnO structure (PDF 005-0664) and Al metal substrate (PDF 089-2769) are included at the bottom for comparison purposes.

The grazing incident XRD pattern of the Al/ZnO sample reveals the formation of nanocrystalline ZnO with a hexagonal structure (space group P63mc) (Figure 2). Additionally, the ZnO coating layer exhibits a preferred (100) orientation during its growth compared to the theoretical pattern (PDF 005-0664) [45]. The average crystallite size of ZnO layer, estimated through the Scherrer equation, is approximately 9.5 nm. On the other hand, diffraction peaks attributed to Al_2_O_3_ are not observed, indicating the predominantly amorphous nature of NPAS component, but a small and narrow peak assigned to Al metal from the Al support is discernible. Previous studies have demonstrated highly textured and oriented ZnO films along the (002) direction, with a crystallite size of 22 nm, when deposited using dc magnetron sputtering on silicon wafers. However, different crystallographic planes were observed when ZnO was deposited on an anodic alumina substrate, showing a somewhat smaller crystallite size (~16 nm) [46]. The variation in the preferred orientation was attributed to the roughness of the alumina substrate. Interestingly, the crystal size of the ZnO coating layer obtained in this work using ALD is slightly smaller than that achieved by magneton sputtering. These findings further confirm that the preferred orientation and resulting crystal size of the ZnO coating layer are highly dependent on the deposition method and substrate morphology. 

**Figure 3:**
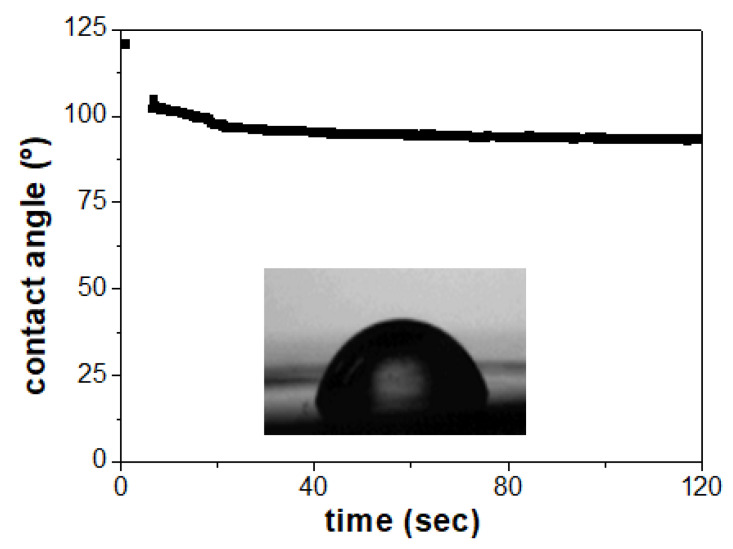
Time evolution of contact-angle measurements for the Al/ZnO nanoporous thin film. A photograph of the water drop is shown in the insert.

Figure 3 shows the time evolution of the contact angle measured for the Al/ZnO film, confirming the constancy of the values after the initial ten seconds. Additionally, a picture of the water drop placed on the surface of the Al/ZnO film shows its highly hydrophobic character. The average value of the film contact angle was (98 ± 6)°, which is higher than that reported for the same NPAS substrate coated by ALD with a SiO_2_ layer (<87 ± 2>° [17]), demonstrating the effect of the ZnO layer on increasing the hydrophobicity of the studied nanostructure. 

### 3.2. Optical Characterization

Information on the optical characteristics of the Al/ZnO nanoporous film was obtained from photoluminescence and spectroscopic ellipsometry measurements, which are two non-destructive and contactless techniques. 

**Figure 4:**
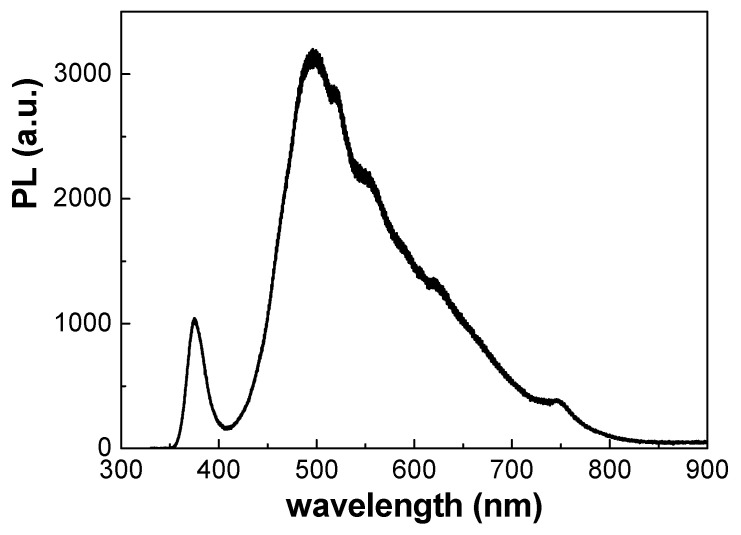
Photoluminescence spectra for the Al/ZnO nanoporous thin film (λ_exc_ = 325 nm).

Photoluminescence (PL) has become a material characteristic of great technological interest, as it has potential applications as a substrate for emitting diodes or sensors [7,8]. In Figure 4, the PL spectra of the Al/ZnO nanoporous film reveals a well-defined narrow peak at 375 nm, which is attributed to ZnO. This value is consistent with those reported for ZnO nanorods (390 nm), ZnO thin layers (372 nm) [31,46,47] or 380 nm in the case of a ZnO layer deposited using the magnetron sputtering technique on a NPAS substrate (with similar pore size/porosity than the studied sample). Additional peaks in the range 430–630 nm were also detected for supported ZnO layers, depending on the deposition conditions and substrates [46,48]. In fact, the PL spectra for nanoporous alumina structures obtained through electrochemical methods exhibit broad maxima in the range of 450–500 nm, depending on the fabrication conditions, such as anodization voltage, time, as well as the type and pH of electrolyte used in the anodic process [7,8]. Consequently, the nanoporous alumina substrate could contribute to the PL signal in this range of wavelengths. 

**Figure 5:**
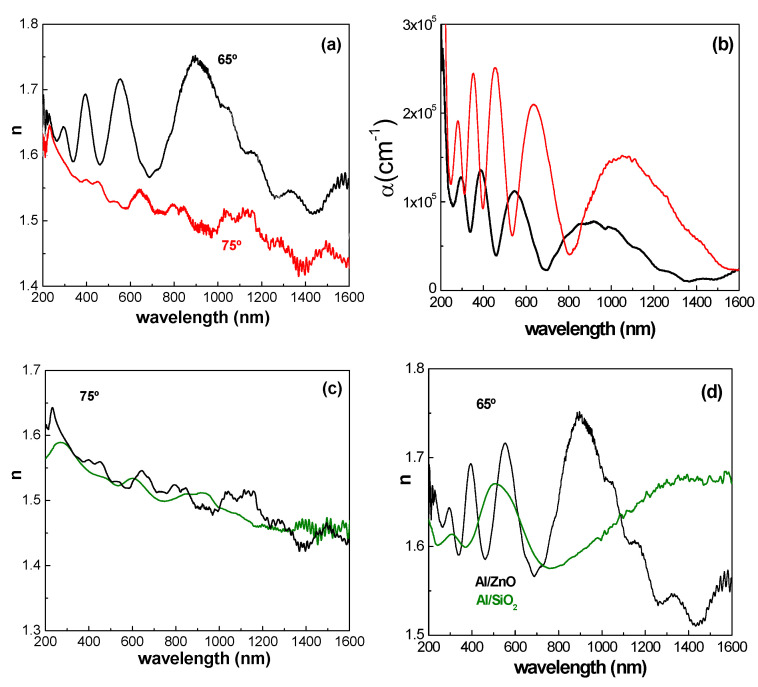
Comparison of wavelength dependence for the Al/ZnO sample of: (**a**) refraction index, and (**b**) absorption coefficient, at two incident angles: 65° (black line) and 75° (red line). Comparison of wavelength dependence of n values for the Al/ZnO sample (black line) and the Al/SiO_2_ sample (green line) at incident angles of 75° (**c**) and 65° (**d**), respectively.

Characteristic optical parameters that are typical for the Al/ZnO nanoporous sample, including the refractive index (n), extinction coefficient (k), and absorption coefficient (α = 4πk/λ, which describes the intensity attenuation of the light passing through a material), were determined from SE experimental parameters (angles Ψ and Δ) by using ellipsometer software v.2.0. The wavelength (λ) dependence of n and α values at the two measured light incident angles (65° and 75°) is shown in Figure 5a,b, while the dependence of Ψ and Δ on λ is shown in the Supplementary Information (Figure S2). Differences in n and α values depending on the incident angle can be observed; these are associated with surface roughness, since it significantly influences the ellipsometric measurements [49,50], and/or superficial contamination (both environmental and that related to sample preparation). 

The composition of the NPAS substrates has been previously examined in different works [19] utilizing XRD and XPS, revealing the presence of amorphous alumina and minimal amounts of aluminum metal. Additionally, it should be highlighted that previous chemical surface characterization of the Al/ZnO sample by XPS [19] showed high atomic concentration percentages of carbon (43.3%), oxygen (37.6%) and aluminum (8.7%), indicating incomplete surface coverage of the NPAS substrate due to the thin ZnO layer. A table with the atomic concentration percentages (AC %) of the different chemical elements present on the surface of the Al/ZnO thin film is provided in Supplementary Information (Table S1). Surface inhomogeneity/contamination effects were also observed in the values of n and α obtained for the same NPAS substrate coated by ALD with a SiO_2_ layer. These later values are rather similar to those reported for ZnO thin films grown on glass substrates by sol–gel spin coating method [51], although as expected, they do not exhibit the oscillatory behavior associated with the nanoporous structure of the Al/ZnO thin film. Figure 5c,d show the values obtained at 65° and 75° incident angles, where differences associated with top surface particularities can also be observed. In any case, the results obtained for Al/ZnO and Al/SiO_2_ samples exhibit the oscillatory character of the refraction index, a characteristic typical of photonic crystals, which has been previously observed for different NPASs coated with different ceramic oxides (with a single layer or two different layers of coverage) [15,19]. 

**Figure 6:**
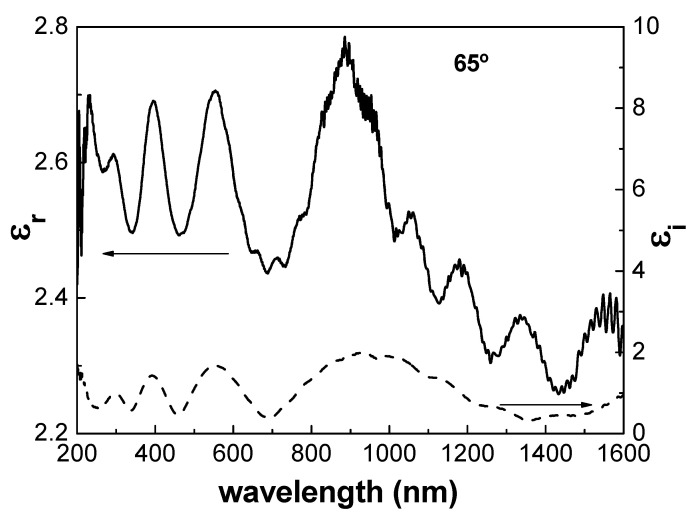
Wavelength dependence of the real (left-side axis) and imaginary (right-side axis) parts of the dielectric constant (ε_r_ or ε_i_) for the Al/ZnO nanoporous thin film from SE measurements performed at 65° incident angle.

From the values of n(λ) and k(λ), the dependence of both the real and imaginary parts of the complex dielectric constant (ε_r_ and ε_i_) for the Al/ZnO nanoporous thin film can be determined, taking into account the relationship: ε = (n + ik)^2^. The wavelength dependence of ε_r_ and ε_i_ values for the Al/ZnO nanoporous sample, at the incident angle of 65°, is shown in Figure 6. Although ε_r_ values also exhibit a noticeable wavelength oscillatory dependence, an average value of 2.55 can be estimated, which is not significantly different from that determined for a ZnO nanostructured thin film obtained by the sol–gel spin coating method on a glass substrate [52]. 

### 3.3. Electrochemical Characterization of the Al/ZnO Nanoporous Film

The electrochemical properties of the Al/ZnO nanoporous thin film in contact with electrolyte solutions can be of great interest for its potential applications as membrane (nanofilter and/or drug delivery system), as previously suggested. Therefore, electrochemical impedance spectroscopy (EIS) measurements were conducted using NaCl solutions at different concentrations to obtain information regarding charge movement/adsorption processes through the Al/ZnO sample, as well as their effects on the electrical properties of both the sample and the solution/sample interface. 

EIS is an alternating current-based technique used to determine the impedance of a system as a function of the current frequency, and it finds widespread application in the electrical characterization of both homogeneous and heterogeneous systems. The impedance, Z(*ω*), is a complex magnitude [Z(ω) = Z_real_ + j Z_img_], where Z_real_ and Z_img_ are the real and imaginary parts, respectively. In homogeneous systems, featuring a unique relaxation process, impedance can be expressed as a combination of resistance (*R*) and capacitance (*C*). The corresponding Nyquist plot (−Z_img_ versus Z_real_) consists of a semicircle, with the maximum occurring at a frequency (f) such that *ωRC* = 1, where ω is the angular frequency (*ω =* 2*πf*). In the case of non-homogeneous systems, the Nyquist plot displays a depressed semicircle, and a constant phase element Q(ω) is then considered, with an equivalent capacitance given by *C_eq_ =* (*RQ*)^1^*^/n^*/*R*, where *n* is an empirical parameter [43]. For composite systems with multiple contributions (i.e., layered solid or solid/liquid systems) involving electrolyte conduction and electrode polarization processes at the electrolyte/sample interface, equivalent circuits comprising a series association of various sub-circuits are commonly used to analyze the impedance data [53,54,55]. 

**Figure 7:**
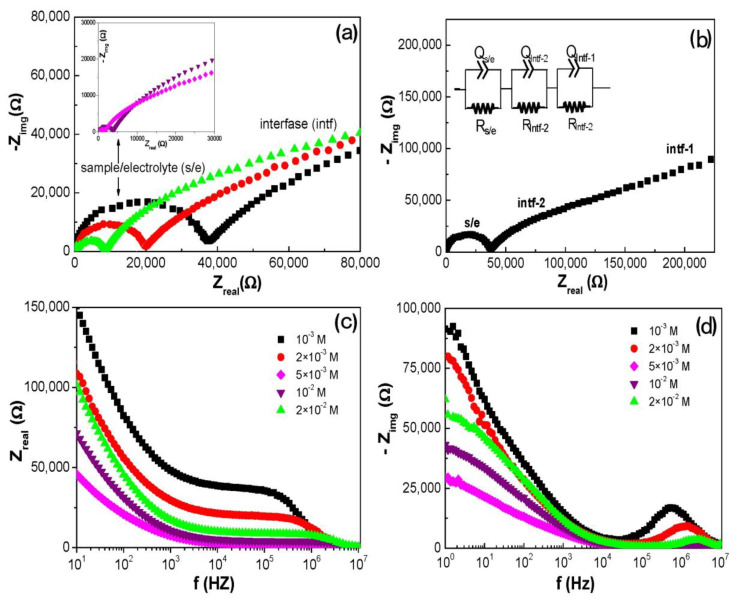
(**a**) Nyquist plots of Al/ZnO nanoporous sample for different NaCl concentrations: 10^−3^ M (■), 2 × 10^−3^ M (●), 5 × 10^−3^ M (▲), 10^−2^ M (▼) and 2 × 10^−2^ M (♦). (**b**) Nyquist plot for 10^−3^ M NaCl blank solution, accompanied by the equivalent circuit used for data fitting. (**c**) Bode plots (Z_real_ vs. frequency) and (**d**) Bode plots (−Z_img_ vs. frequency).

Figure 7a presents the Nyquist plots obtained for the Al/ZnO nanoporous thin film when in contact with NaCl solutions of various concentrations. Two different contributions are clearly discernible: (i) the Al/ZnO thin film with its nanopores filled with the electrolyte solution, along with the electrolyte solution between the film surface and the Pt electrodes (s/e contribution); (ii) the electrolyte/film interfacial contribution (intf). In all cases, Nyquist plots show a slightly depressed semicircle at high frequency attributed to both the electrolyte and the Al/ZnO nanoporous thin film (s/e) with similar relaxation frequencies (one associated with the film and the other with the electrolyte). The size of the semicircle decreases with the increase in NaCl concentration due to the increase in the solution conductivity. It is worth noting that the total impedance data for the entire frequency range, with each solution concentration, are not displayed in Figure 7a for scaling reasons, due to the high interfacial contribution exhibited by the electrolyte–Al/ZnO nanoporous film system. Nevertheless, they can be observed in Figure 7b for 10^−3^ M NaCl solution data, and in the Bode plots presented in Figure 7c (Z_real_ vs. frequency) and Figure 7d (−Z_img_ vs. frequency). This latter picture also shows the shift to a higher frequency of the peak associated with the sample/electrolyte contribution as a result of an increase in the solution concentration, resembling those observed for the electrolyte solutions [54]. 

In fact, the minimal effect of the Al/ZnO nanoporous sample on the impedance values of the *s/e* contribution, along with its high interfacial contribution, is evident when comparing the impedance plots for the Al/ZnO sample in contact with a 2·10^−3^ NaCl solution with those for the solution alone (without any sample in the measuring cell), or for an Al/SiO_2_ nanoporous film (SiO_2_ coating layer achieved by ALD on the same alumina substrate) provided as Supplementary Information (Figure S3) for comparison purposes. As observed, the presence of the Al/ZnO sample significantly affects the interfacial values, possibly due to its stronger hydrophobic character compared to the analogous sample with SiO_2_ coating; additionally, it influences the peak position frequency, shifting to a higher frequency (from 1.1 MHz for the sample/electrolyte system to 2.5 MHz for the electrolyte system). In fact, lower interfacial contribution (Donnan exclusion) for the Al/SiO_2_ nanoporous film compared to the Al/ZnO one was also obtained from concentration or membrane potential measurements already performed with NaCl solutions [19].

**Figure 8:**
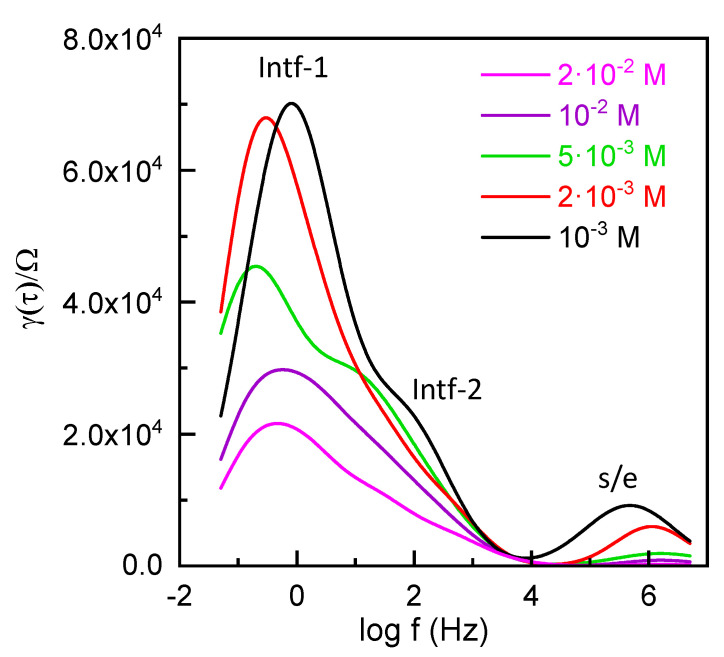
DRT curves for the Al/ZnO nanoporous film when immersed in NaCl solution at different concentrations.

Figure 7b also displays two distinct interfacial contributions (intf-1 and intf-2). Subsequently, a series association of three different sub-circuits (RQ elements) is considered to represent the Al/ZnO nanoporous sample combined with the electrolyte solution system. To confirm these contributions, the Distribution of Relaxation Time (DRT) method was employed. This method involves transforming the spectra from the frequency domain to the time domain and is considered a model-free approach to characterizing resistive–capacitive systems. It identifies the number of electrochemical processes involved without relying on prior assumptions and is commonly used for the characterization of fuel cells and batteries [56]. Figure 8 shows the DRT curves for the Al/ZnO nanoporous sample at different NaCl concentrations, clearly revealing two different interfacial processes at low frequency that support the three contributions indicated in the equivalent circuit of the inset Figure 7b.

**Figure 9:**
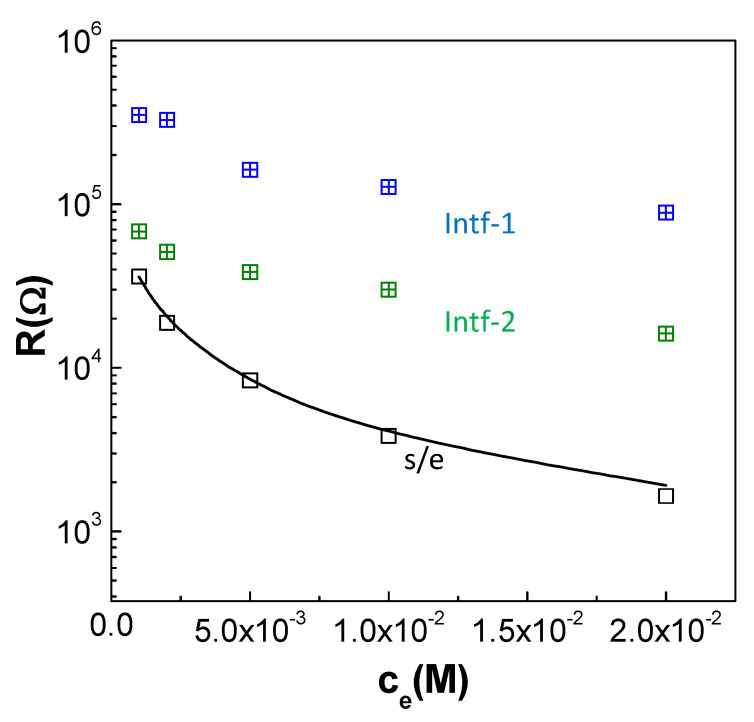
Variation in the electrical resistance of the sample/electrolyte (s/e) and the interfaces (intf) with the electrolyte concentration. The electrical resistance of the NaCl solutions is included as a solid line.

The fitting of the experimental data to the proposed equivalent circuit allows for the estimation of the electrical resistance and capacitance associated with the sample/electrolyte (s/e), as well as the interfacial effects intf-1 and intf-*2*, which are typically linked to the double-layer capacitance and the charge transfer processes at the electrolyte/sample interfaces. Figure 9 compares the resistance contributions at different NaCl solution concentrations. Furthermore, a solid line representing the electrical resistance of a reference NaCl solution (determined considering solution conductivity and the measuring cell constant) is included for comparison. As expected, the sample/electrolyte resistance decreases with the NaCl concentration due to higher electrical conductivity, while the capacitance remains nearly constant, with typical values of 10^−11^ F. The Al/ZnO nanoporous film has a minimal impact on the overall resistance of the s/e contribution, which is comparable to that for NaCl solutions. On the other hand, a similar trend is observed for the interfacial processes associated with the double-layer capacitance and charge transfer processes at the electrolyte/sample interfaces reported for membranes in contact with electrolyte solutions [57]. While these results highlight the negligible impact of the Al/ZnO sample on the overall s/e resistance, they also underscore the influence of surface material on the electrochemical processes occurring at the sample/electrolyte interface. 

## 4. Conclusions

A nanoporous alumina structure (NPAS) with a high aspect ratio (~3000), synthesized through a two-step electrochemical anodization process, featuring well-defined and parallelly aligned pore channels and a thickness of around 60 µm, was functionalized with a ZnO coating layer (~5 nm in thickness) using ALD. The ZnO coating not only reduced the pore diameter (~18 nm) and porosity of NPAs but also substantially modified their optical and electrochemical properties. XRD analysis indicated that the ZnO layer exhibited a preferred (100) orientation with an average crystallite size of approximately 9.5 nm, effectively reducing the hydrophobicity of the alumina substrate, as evidenced by contact-angle measurements.

The effect of the ZnO coating layer on characteristic optical parameters such as the refraction index, the absorption coefficient and the dielectric constant, as well as the effect of surface peculiarities (roughness/contamination), was determined using spectroscopic ellipsometry. These findings revealed the photonic crystal nature of the Al/ZnO nanoporous thin film. Moreover, PL analysis demonstrated a dominant signal at ~375 nm, consistent with the successful deposition of the ZnO layer. On the other hand, electrochemical impedance spectroscopy measurements clearly confirmed the effect of ZnO surface modification on the electrochemical processes occurring at the sample/electrolyte (NaCl) interface. These results underscore the potential for tailoring the optical and interfacial properties of NPASs by selecting the most suitable coating material for specific applications, particularly technological fields where precise control over optical and interfacial characteristics is essential.

## Data Availability

Data are contained within the article and Supplementary Materials.

## Supplementary Materials

The following supporting information can be downloaded at: https://www.mdpi.com/article/10.3390/ma17061412/s1, Figure S1: Scheme of dead-end electrochemical cell; Figure S2: Wavelength dependence of spectroscopy ellipsometry experimental angles; Figure S3: Comparison of impedance plots for samples Al-ZnO and Al-SiO_2_, and the NaCl solution; Table S1: Atomic concentration percentages of the elements found on the surface of the Al/ZnO thin nanofilm.
