# Supplementary material for: Optical and Electrochemical Properties of a Nanostructured ZnO Thin Layer Deposited on a Nanoporous Alumina Structure via Atomic Layer Deposition
Source: Materials (Basel). 2024 Mar 20;17(6):1412. doi: 10.3390/ma17061412 (PMC10972226; doi:10.3390/ma17061412)
Supplement: Supplementary file 1 [file materials-17-01412-s001.zip › materials-2901765-supplementary.pdf]

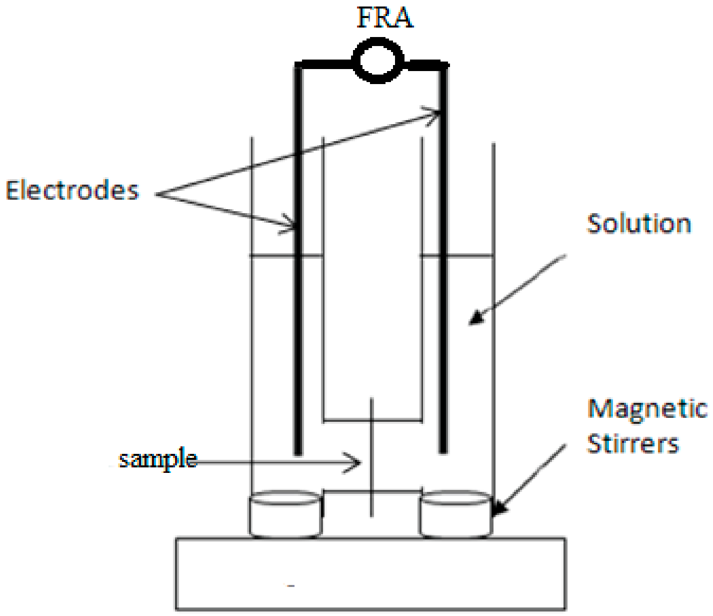

Figure S1. Scheme of dead-end electrochemical cell [44].

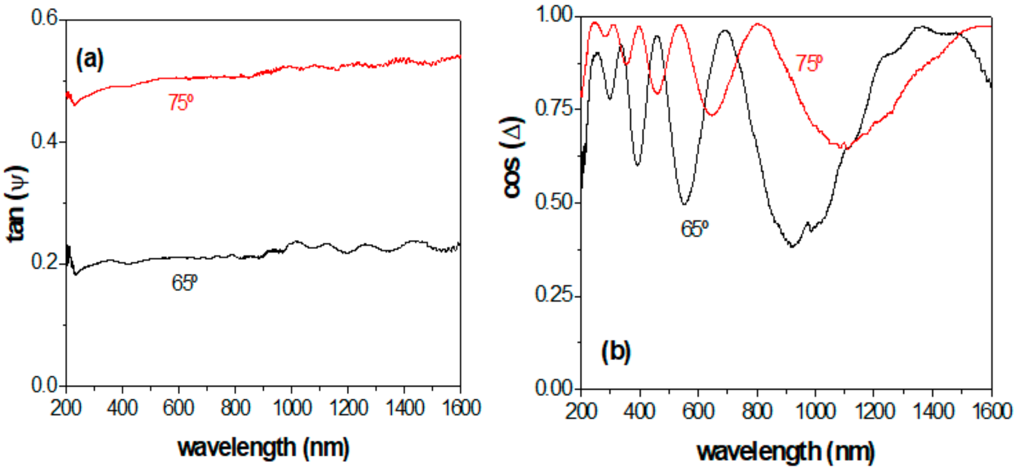

Figure S2. Wavelength dependence of spectroscopy ellipsometry experimental angles: (a) tan(Ψ); (b) cos(Δ), for the two measured angles (65º and 75º).

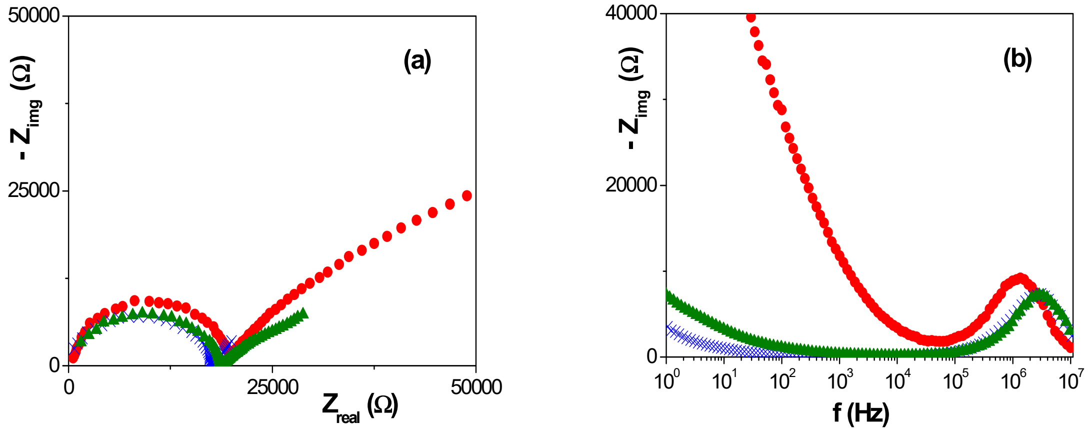

Figure S3. Comparison of impedance plots for Al-ZnO (●) and Al-SiO_2 (▲) samples in contact with $2x10^{-3}$ M NaCl solution, and the $2x10^{-3}$ M NaCl solution (x). (a) Nyquist plots; (b) Bode plots. Data values for Al-ZnO sample starts at 30 Hz for scaling reason.

Table S1: Atomic concentration percentages of the elements found on the surface of the Al/ZnO thin nanofilm*.

Sample	C (%)	Al (%)	O (%)	Zn (%)
NPAS+ZnO	43.3	8.7	37.6	10.2

* elements with A.C. % ≤ 0.5 are not indicated.
